# Heterologous expression of nattokinase from *B. subtilis* natto using *Pichia pastoris* GS115 and assessment of its thrombolytic activity

**DOI:** 10.1186/s12896-021-00708-4

**Published:** 2021-08-09

**Authors:** Yan Guangbo, Shu Min, Shen Wei, Ma Lixin, Zhai Chao, Wang Yaping, Huang Zunxi

**Affiliations:** 1 State Key Laboratory of Biocatalysis and Enzyme, Engineering Hubei Collaborative Innovation Center for Green Transformation of Bio-Resources, Hubei Key Laboratory of Industrial Biotechnology, Biology Faculty of Hubei University, Hubei University, Wuhan, Hubei Province 430062 People’s Republic of China; 2 School of Life Sciences, Yunnan Normal University, Kunming, Yunnan People’s Republic of China

**Keywords:** Nattokinase; High-density fermentation; Multi-copy strains; Pichia pastoris

## Abstract

**Background:**

Nattokinase is a fibrinolytic enzyme that has huge market value as a nutritional supplement for health promotion. In order to increase nattokinase yields, fermentation conditions, strains, cultivation media, and feeding strategies have been optimized. Nattokinase has been expressed using several heterologous expression systems. *Pichia pastoris* heterologous expression system was the alternative.

**Results:**

This report aimed to express high levels of nattokinase from *B. subtilis* natto (NK-Bs) using a *Pichia pastoris* heterologous expression system and assess its fibrinolytic activity in vivo. Multicopy expression strains bearing 1–7 copies of the *apr*N gene were constructed. The expression level of the target protein reached a maximum at five copies of the target gene. However, multicopy expression strains were not stable in shake-flask or high-density fermentation, causing significant differences in the yield of the target protein among batches. Therefore, *P. pastoris* bearing a single copy of *apr*N was used in shake-flask and high-density fermentation. Target protein yield was 320 mg/L in shake-flask fermentation and approximately 9.5 g/L in high-density fermentation. The recombinant nattokinase showed high thermo- and pH-stability. The present study also demonstrated that recombinant NK-Bs had obvious thrombolytic activity.

**Conclusions:**

This study suggests that the *P. pastoris* expression system is an ideal platform for the large-scale, low-cost preparation of nattokinase.

**Supplementary Information:**

The online version contains supplementary material available at 10.1186/s12896-021-00708-4.

## Background

Nattokinase (E.C 3.4.21.62) was first found in natto, a traditional Japanese fermented soybean food product [1]. Full length nattokinase includes 381 amino acids and is encoded by the *apr*N gene of *B. subtilis* natto [2]. After removal of the 29-amino acid signal peptide and 77-amino acid propeptide from the precursor, the mature nattokinase consists of 275 amino acids and is 27.7 kDa with an isoelectric point (pI) of pH 8.6 [2]. Nattokinase is a serine protease, and its activity is strongly inhibited by phenylmethylsulfonyl fluoride (PMSF). Because the enzyme is a cysteine-free proteinase, no disulfide bonds are observed on its structure [2]. A three-dimensional structural model of nattokinase from *B. subtilis* natto (NK-Bs) was constructed using homology modeling in 2005, and the crystal structure of the enzyme was published later (PDB: 4DWW, 3vyv) [3–5]. The catalytic center of nattokinase consists of Ser-His-Asp (D32, H64, S221), and Ser221 is the key residue for substrate recognition.

Nattokinase has been extensively studied because of its unique features as a fibrinolytic enzyme. Although nattokinase shows high similarity to many subtilisins in the serine protease family, it is the only member with thrombolytic activity. Nattokinase can directly cleave cross-linked fibrin in vitro and in vivo [6–8]. Moreover, it contributes to fibrin degradation by inactivating the fibrinolysis inhibitor PAI-1 [9] and catalyzing the conversion of pro-urokinase to urokinase. Furthermore, it stimulates cells to release the tissue plasminogen activator t-PA and decreases plasma levels of fibrinogens. More importantly, unlike common fibrinolytic proteases such as t-PA and streptokinase, nattokinase has a long half-life in blood vessels and causes few side effects, such as bleeding or gastric ulcers. These characteristics make nattokinase a promising drug for the prevention and treatment of cardiovascular diseases. Currently, nattokinase products are used worldwide in the form of tablets or capsules as nutritional supplement for health promotion, including blooding thinning, blood clot prevention, and improving blood circulation [10–12].

Nattokinases are ideal for use in the clinic owing to the low costs associated with their industrial production. Nattokinase is obtained mainly through microbial fermentation, and nattokinase-producing strains have been isolated from natto, douche, and doen-jang. In order to increase nattokinase yields, fermentation conditions, strains, cultivation media, and feeding strategies have been optimized [13–18]. With the development of gene engineering, nattokinase has also been expressed using several heterologous expression systems, such as the multiple protease-deficient host strains *B. subtilis* WB800, *Escherichia coli, Lactococcus lactis, Bacillus licheniformis,* and *Spodoptera frugiperda* [19–24]*.* Our previous study indicated that the *Pichia pastoris* expression system is an ideal system for recombinant expression of protease, which is toxic to cells and causes cell lysis when large numbers of active enzyme molecules accumulate in cells. The tightly regulated *AOX*1 promoter prevents leaky expression while secretory expression of the target protease alleviates the stress proteases cause to cells. Therefore, this system was chosen to obtain high-level expression of nattokinase. The results of the present study indicate that nattokinase from *B. subtilis* natto is able to be expressed at high titers using the *P. pastoris* system, and the recombinant nattokinase exhibits obvious thrombolytic activity along with strong pH- and thermo-stability.

## Results

### Multicopy expression of NK-Bs using *P. pastoris* GS115

**Fig. 1:**
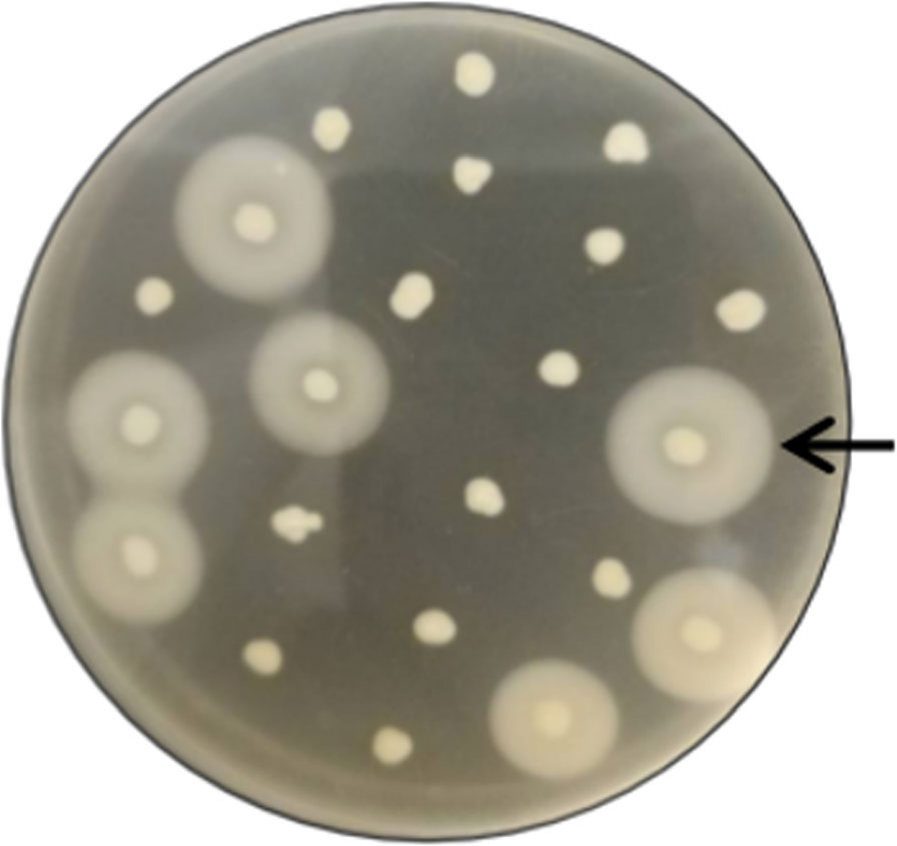
Identification of positive *P. pastoris* recombinants expressing nattokinase from *B. subtilis* natto using a plate assay. The arrow indicates a positive recombinant. Scale bar, 1 cm

The recombinant plasmid was named pHBM905BDM-NK-Bs, transformed into *P. pastoris* GS115, and selected on MD plates without histidine. Twenty-two positive colonies were tested on BMMY media supplemented with 1% (w/v) of casein. After induction with methanol for 48 h, opaque halos appeared around seven colonies (Fig. 1), indicating that nattokinase from *B. subtilis* natto was successfully expressed by these colonies. These recombinants were named NK-Bs-1.

**Fig. 2:**
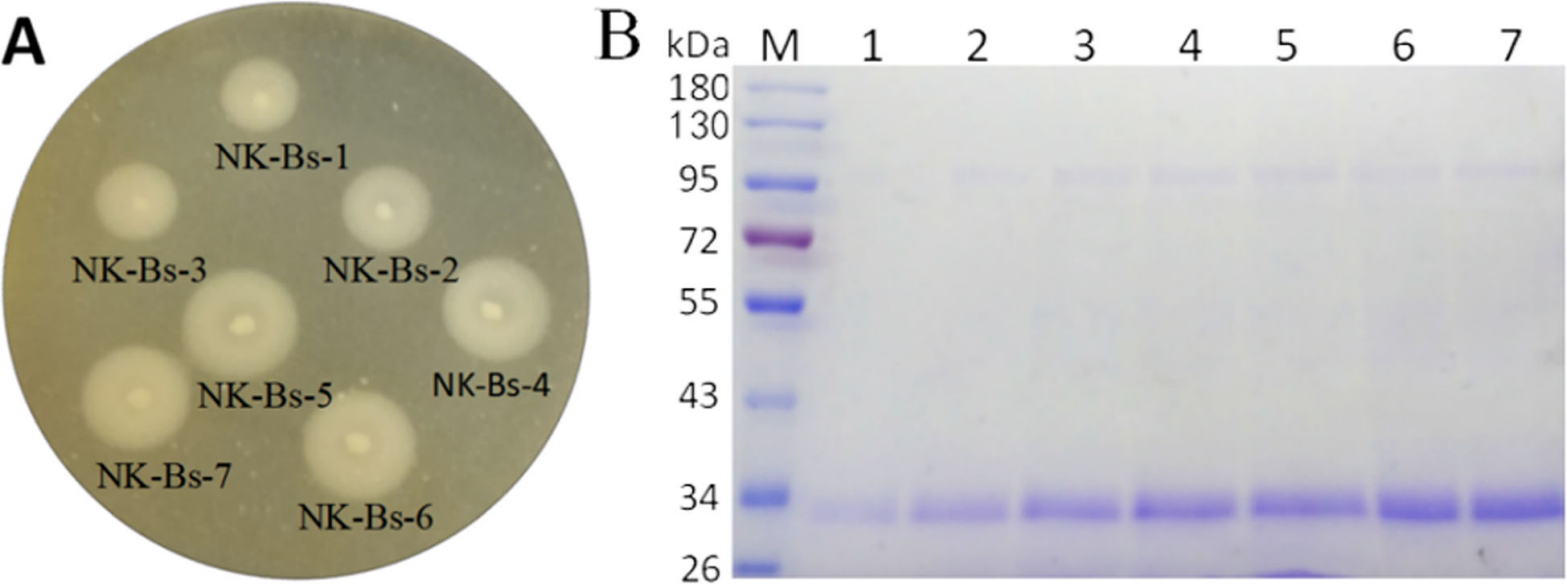
Correlation between recombinant protein titer and gene copy number. **A** The relationship between NK-Bs expression levels and *apr*N gene copy number were assessed using a plate assay. The copy number of the target gene in each strain is indicated below each colony. The circle around each colony represents the average halo size of responding multicopy expression strains. Scale bar, 1 cm. **B** SDS-PAGE analysis of NK-Bs secreted into the culture supernatant. M: protein molecular weight marker (the molecular weight of each band is indicated on the left); lanes 1–7: supernatants from shake-flask fermentation of NK-Bs 1–7 strains, respectively, collected after an 84-h induction with methanol. A total of 20 μL of each sample was loaded

To improve target protein yield, multicopy expression vectors bearing two to seven copies of the NK-Bs expression cassette were constructed. The vectors were linearized and transformed into *P. pastoris* GS115. Positive transformants were named NK-Bs-2 to NK-Bs-7. NK-Bs-1 to NK-Bs-7 strains were inoculated onto BMMY plates supplemented with casein. After two days, a clear, opaque halo formed around each colony. The diameter of the halos around the colonies increased significantly with increasing target gene copy number, suggesting a correlation between recombinant protein titer and gene copy number (Fig. S1). The size of the halo, indicating the effect of the gene copy number, reached a maximum at five copies (Fig. 2A, Table S2). Expression levels of the target protein in the multicopy expression strains were also analyzed using shake-flask fermentation. SDS-PAGE revealed a main band of approximately 34 kDa after 84 h of induction, and the bands became more obvious as the number of gene copies increased (Fig. 2B). These results indicate that higher gene copy numbers improved nattokinase yield.

### Shake-flask and high-density fermentation of NK-Bs

**Fig. 3:**
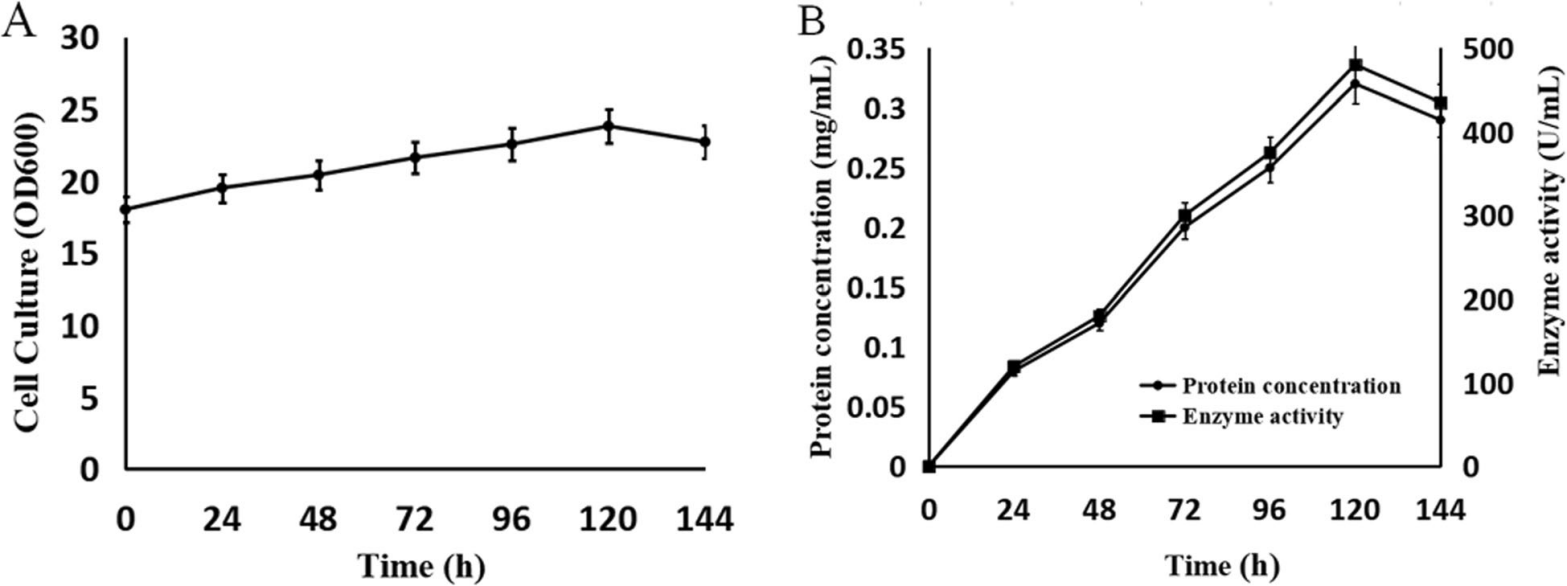
Quantitative analysis of the expression of NK-Bs produced by shake-flask fermentation. **A** Time course of cell density during the induction phase. **B** Time course of the titer of the target protein and serine protease activity

**Fig. 4:**
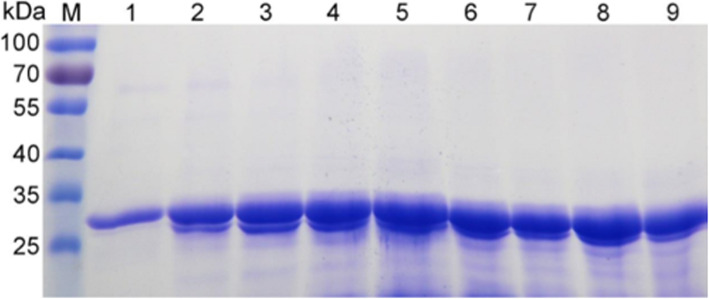
SDS-PAGE analysis of NK-Bs secreted into the supernatant during high-density fermentation. M: protein molecular weight marker (the molecular weight of each band is indicated on the left); lanes 1–9: cell culture supernatant after induction with 1% methanol for 12, 24, 36, 48, 60, 72, 84, 96, and 108 h, respectively

Further study indicated that the multicopy expression strains were not stable during shake-flask or high-density fermentation, causing remarkable differences in the titer of the target protein among batches. Therefore, to ensure the reliability of the results, strain NK-Bs-1 was used for all following experiments. With shake-flask fermentation, the OD_600_ of the cell culture was approximately 18 at the beginning of the induction and reached a maximum of approximately 24 after 120 h (Fig. 3A). The protein concentration and enzyme activity of the recombinant protein were associated with cell growth and reached 0.32 g/L and 480 U/mL, respectively, after 120 h (Fig. 3B). In high-density fermentation, the yield of the target protein reached a maximum of approximately 9.5 g/L and 15,421 U/mL after 96 h (Fig. 4).

### The characteristics of NK-Bs

**Fig. 5:**
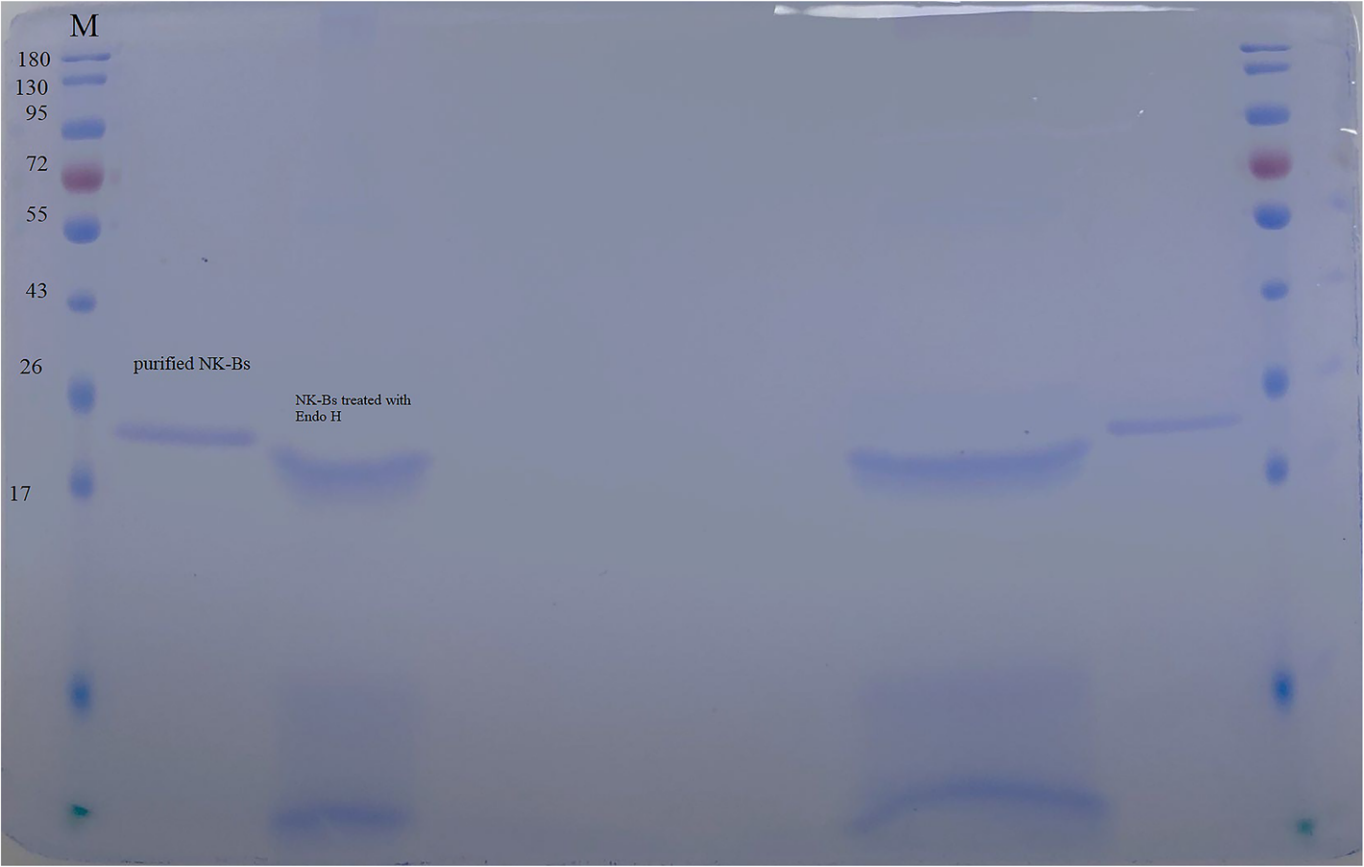
Purification of NK-Bs. **A** SDS-PAGE of purified NK-Bs. M: protein molecular weight marker (the molecular weight of each band is indicated on the left). Lane 1: supernatant from shake-flask fermentation. Lane 2: target protein purified with Ni^2+^-affinity chromatography. **B** Glycosylation analysis of NK-Bs. Lane 1: purified NK-Bs. Lane 2: NK-Bs treated with Endo H

**Fig. 6:**
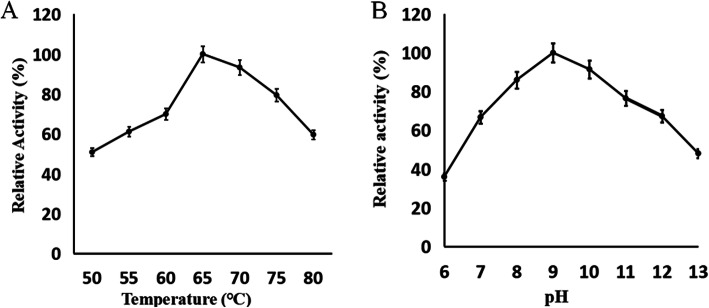
The optimal temperature and pH for NK-Bs. **A** The optimal temperature for NK-Bs. The enzyme activity at 65 °C was set to 100%. The absolute enzyme activity, corresponding to 100% activity, was 465 U/mL. **B** The optimal pH for NK-Bs. The enzyme activity at pH 9.0 was set to 100%. The absolute enzyme activity, corresponding to 100% activity, was 371.7 U/mL

**Fig. 7:**
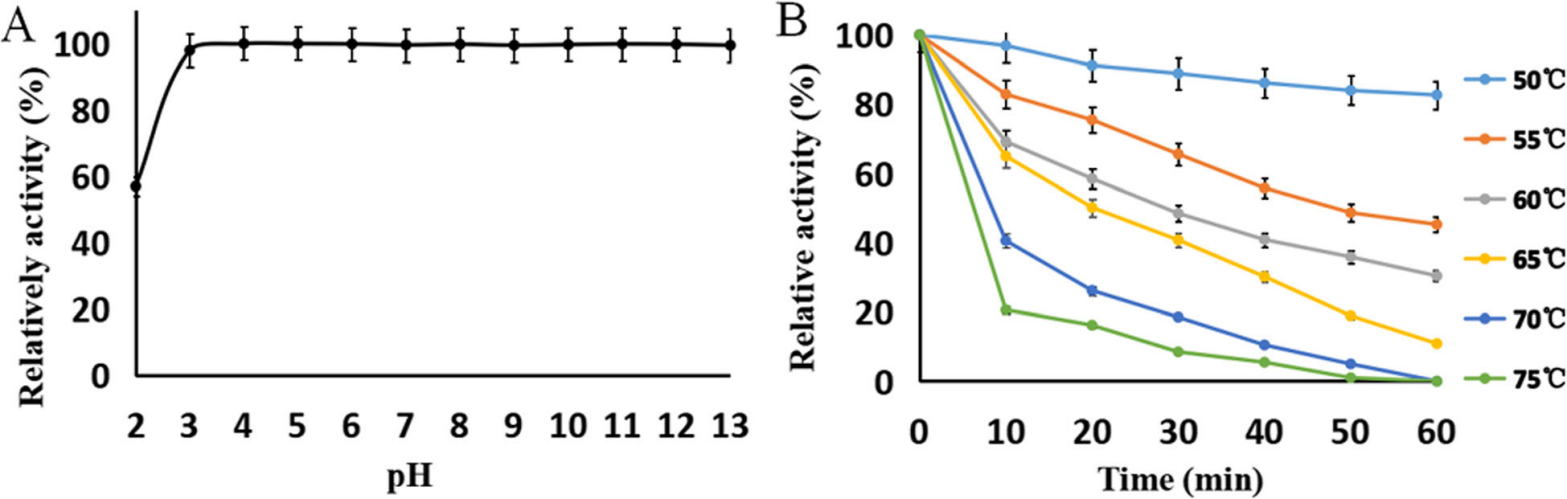
Effect of pH and temperature on the stability of NK-Bs. **A** pH stability of NK-Bs. **B** Thermostability of NK-Bs at 50–75 °C, at increments of 5 °C

To study the characteristics of NK-Bs, the target protein was purified and concentrated (Fig. 5A). The recombinant NK-Bs was slightly larger than predicted. After Endo H treatment, the size of NK-Bs decreased to approximately 27 kDa, which was consistent with the predicted size of nattokinase (Fig. 5B). NK-Bs had high activity within a broad temperature and pH range. The optimal pH of the enzyme was pH 9.0, and approximately 80% of its activity was retained at pH 8.0–10.0 (Fig. 6B). The optimal temperature of NK-Bs was 65 °C, and over 80% of its activity was retained at 75 °C (Fig. 6A). NK-Bs exhibited outstanding stability at a wide range of pH values and retained almost full activity after incubation at pH 3.0–13.0 for 1 h (Fig. 7A). NK-Bs also had relatively high thermostability (Fig. 7B). NK-Bs retained over 50 and 40% of its enzymatic activity after incubation for 30 min at 60 °C and 65 °C, respectively. However, its activity decreased dramatically above 65 °C, and its half-life was less than 10 min at 70 °C.

The effects of chemical reagents and metal ions on the activity of NK-Bs were investigated. Because there is no cysteine in NK-Bs, β-mercaptoethanol had no obvious effect on the enzymatic activity (Table 1). Similarly, ethylenediaminetetraacetic acid (EDTA) had no obvious effect on the enzyme activity of NK-Bs (Table 1), indicating that NK-Bs is not a metalloprotease. However, PMSF dramatically inhibited enzyme activity (Table 1), which is consistent with a previous report that NK-Bs is a serine protease. Heavy metal ions, such as Fe^3+^ and Cu^2+^, significantly inhibited the activity of NK-Bs at both 1 and 5 mM. Similarly, Zn^2+^, Fe^2+^, Ni^2+^, and Ba^2+^ inhibited the enzymatic activity at 5 mM. In contrast, Mg^2+^, Mn^2+^, and Ca^2+^ had no obvious effects on the activity of the recombinant protein at either concentration (Table 2).

**Table 1:** the effect of protease inhibitors on the activity of NK-Bs

inhibitor	residual activity (%)	
	1 mM	2 mM
*β*-mercaptoethanol	97.48 ± 3.72	92.90 ± 3.51
EDTA	109.46 ± 2.06	105.11 ± 4.50
PMSF	40.17 ± 1.84	3.02 ± 0.36
control	100.00 ± 1.62	100.00 ± 1.27

**Table 2:**
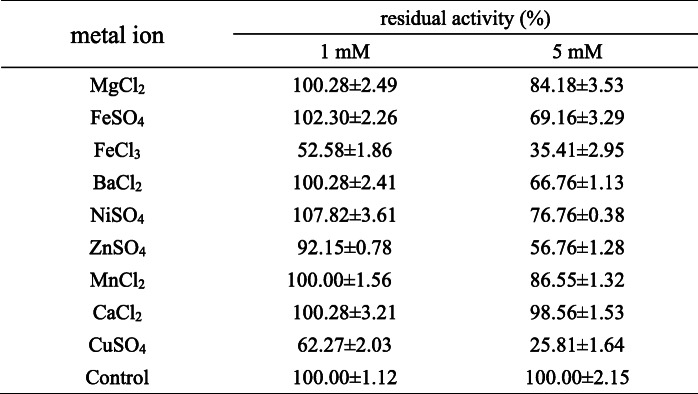
the effect of metal ions on the activity of NK-Bs

### The thrombolytic/fibrinolytic effect of NK-Bs in vitro

**Fig. 8:**
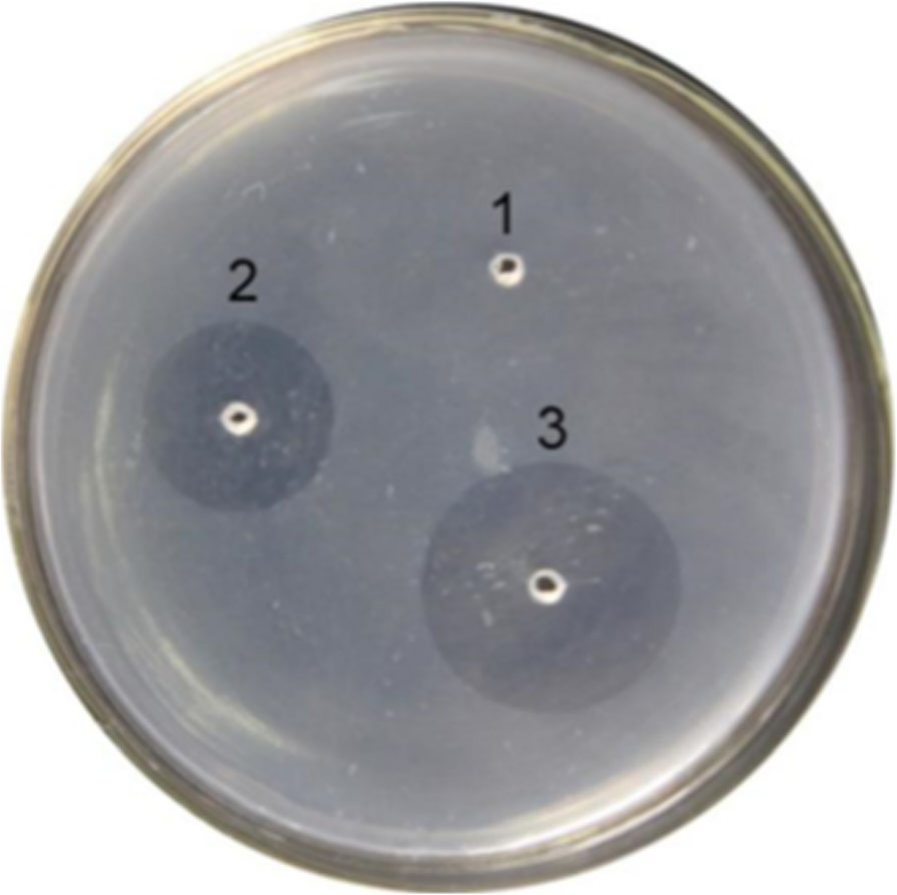
Analysis of the thrombolytic activity of NK-Bs using fibrin plates. 1: negative control (10 μL physiologic saline); 2: positive control (10 μL commercial lumbrokinase); 3:10 μL NK-Bs. scale bar, 1 cm

The fibrinolytic activity of NK-Bs was tested using fibrin plates. A clear halo appeared around NK-Bs and commercial lumbrokinase, which was applied as a positive control (Fig. 8). These results demonstrated that the recombinant NK-Bs had high fibrinolytic activity.

### The thrombolytic/fibrinolytic effect of NK-Bs in vivo

**Fig. 9:**
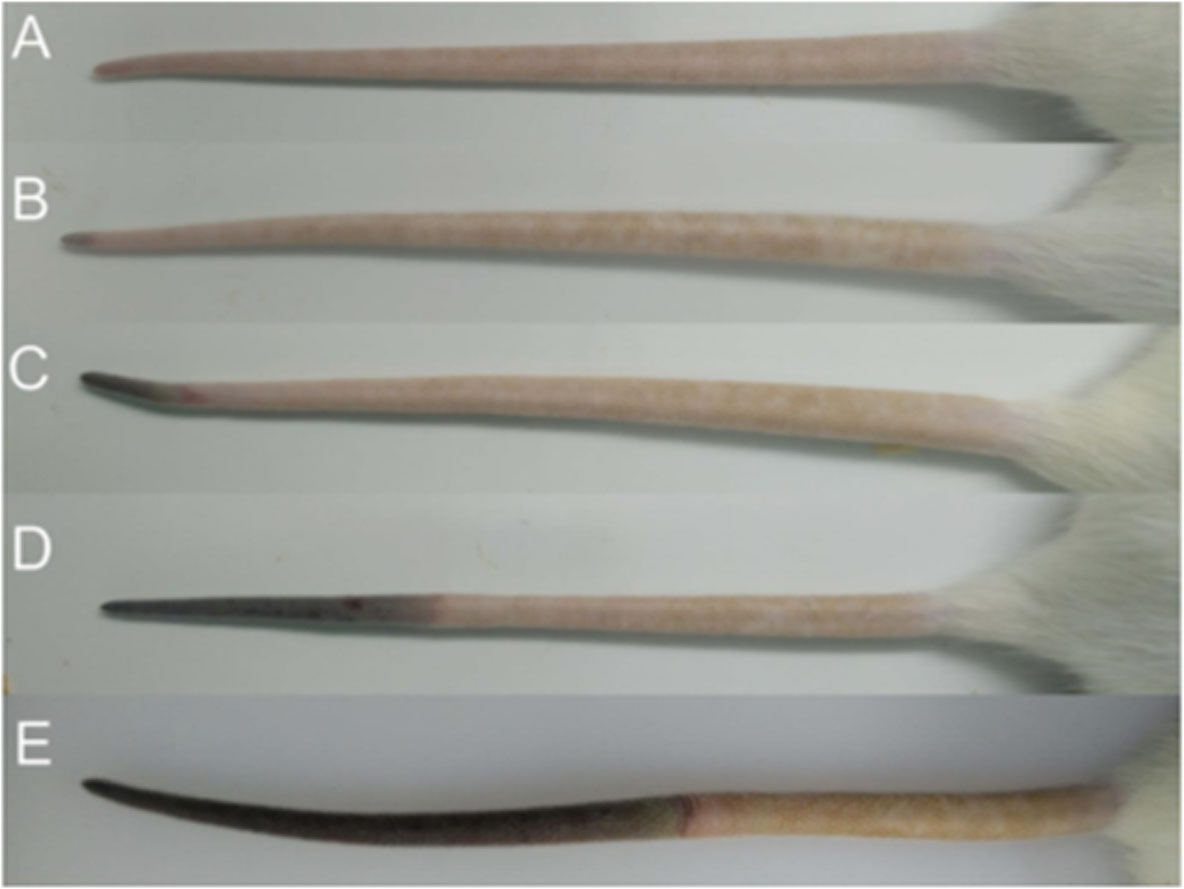
Infarction of the tips of the tails of rats injected with κ-carrageenan. **A**Control group; **B** 4 h post-injection; **C** 8 h post-injection; **D** 12 h post-injection; **E** 24 h post-injection. Scale bar, 0.5 cm

**Fig. 10:**
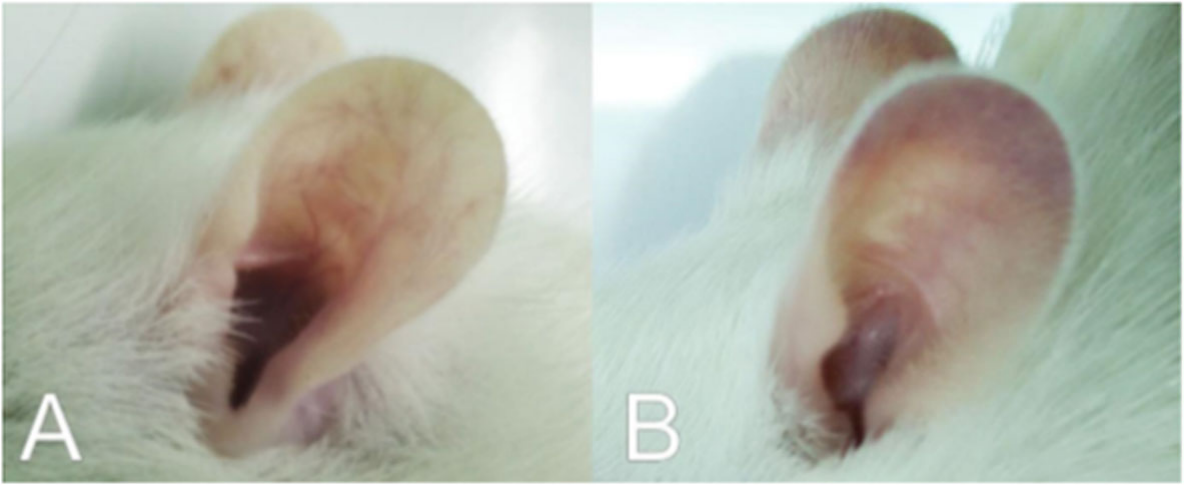
Ears of rats (**A**) before and (**B**) 24 h after injection with κ-carrageenan

Straight-chained, sulfur-containing polysaccharide κ-carrageenan was used to construct a rat model of microvascular thrombosis. The dosage of κ-carrageenan used for injection was 2.0 mg/mL and the frequency of tail infarction was 85%. After 4 h, tail tips became black. Tail infarction was visible after 8 h and lengthened after 12 h (Fig. 9). The ears of the rats turned black 24 h after injection (Fig. 10).

**Fig. 11:**
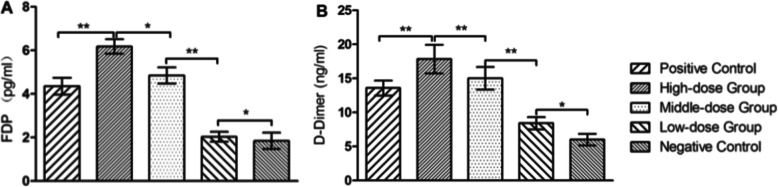
Changes in plasma concentrations of FDPs and D-dimer after nattokinase treatment. According to the concentration of the standard, we calculated the FDP and D-dimer concentrations using the OD value of the samples. * and ** indicate statistically significant differences at *P* ≤ 0.05 and *P* ≤ 0.01, respectively. **A** Changes in plasma concentrations of FDPs of rats that treated by different levels of NK-Bs. **B**Changes in plasma concentrations of D-dimer of rats that treated by different levels of NK-Bs

**Fig. 12:**
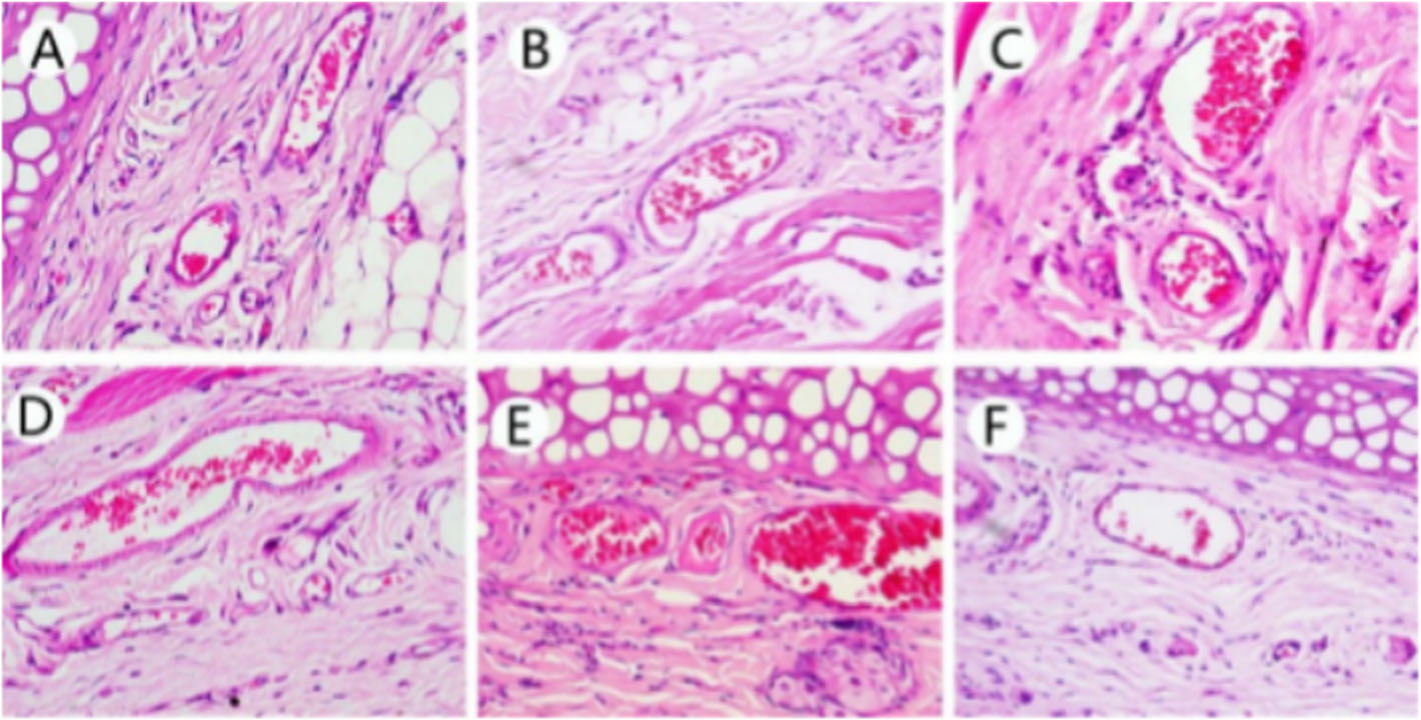
Tissue biopsies of the ears of thrombotic rats treated with different doses of NK-Bs. **A** High dose of NK-Bs; **B** medium dose of NK-Bs; **C** low dose of NK-Bs; **D** positive control (treated with vermis kinase); **E** negative control (treated with physiologic saline); **F**rats not given κ-carrageenan treatment. Scale bar, 100 μm

After the thrombosis rat model was constructed, the rats were treated with different doses of nattokinase for 1 week, and FDPs and D-dimer levels in blood samples were analyzed. The plasma concentrations of FDP and D-dimer of rats that received a high or medium dose of NK-Bs were similar to those treated with vermis kinase but were significantly higher than the negative control. In contrast, the concentrations of FDP and D-dimer of rats that received a low dose treatment were similar to the negative controls (Table 3, Fig. 11). In addition, ear tissue was examined histologically. Tissue slices of normal rat ears displayed no obvious thrombosis in the lumen (Fig. 12F), whereas thrombosis was evident in the entire lumen in thrombotic rats (Fig. 12E). However, after treatment with nattokinase, the thrombosis in the lumen was significantly reduced (Fig. 12A-C). Furthermore, the reduction became more obvious as the dose of NK-Bs increased. The effect in the high dosage group was similar to that of rats treated with vermis kinase (Fig. 12D). These results indicate that the recombinant nattokinase had an obvious fibrinolytic effect, and this effect was dose dependent.

**Table 3:** The physiological parameters of rats after the nattokinase treatment

groups of rats	dosage of enzymes (IU/kg)	FDP (pg/mL)	D-dimer (ng/mL)
Positive control (vermis kinase)	10,000	5.12 ± 0.31	15.02 ± 2.36
High dosage	50,000	6.17 ± 0.32	17.85 ± 2.10
Medium dosage	10,000	4.36 ± 0.21	13.59 ± 1.29
Low dosage	2000	2.04 ± 0.06	8.42 ± 1.88
Negative control (physiologic saline)	0	1.84 ± 0.27	6.00 ± 0.87

* FDP: Fibrin/fibrinogen degradation products (FDP) is the general term for fibrin degradation products and fibrinogen degradation products, including a variety of peptides of different molecular weights.

D-dimer: The fibrin monomer was first cross-linked by activated factor XIII and then hydrolyzed by plasmin. The final products were D-dimer.

## Discussion

*P. pastoris* is generally regarded as safe (GRAS). In the present study, we expressed nattokinase, which has great potential as a healthcare product, using a *P. pastoris* secretary expression system [24–27]. In 2005, nattokinase was expressed in *E. coli* with oleosin fused to its N- or C-terminus, yielding 300 mg/L of nattokinase with a recovery yield of 60% [23]. In 2013, nattokinase was also expressed in *B. subtilis* with a titer of 600 mg/L, which was the highest expression level achieved to date [18]. Luo et al., expressed nattokinase using *P. pastoris* and obtained a target protein titer much lower than by heterologous expression in prokaryotes and original *Bacillus* strains [28]. There are two reasons that we significantly increase the productivity of producer strains. Firstly, the aprN gene was optimized based on the codon preference of P. pastoris and then synthesized. But Luo cloned the nattokinase gene directly from B.subilit and inserted into the pPICZαA vector. The naturel nattokinase gene maybe not appropriate expressed in *P.pastoris*. Secondly, we used vector pHBM905BDM which has a dl + 2 × 201 AOX1 and a MF4I leader sequence. This strong dl + 2 × 201 AOX1 promoter could significantly improved the protein express level. However, Luo only used the original pPICZαA vector which has only a AOX1 promoter. In comparison with these reports, the maximum expression level of nattokinase in the present study was 320 mg/L with shake-flask fermentation and 9.5 g/L with high-density fermentation. Moreover, the recombinant nattokinase displayed remarkable thermo- and pH-stability. Consistent with the previous report, the NK-Bs produced in the present study had over 60% activity at pH 7.0–11.0. Previous reports have indicated that acidic conditions cause a rapid decease in the enzyme activity of nattokinases, whereas the recombinant NK-Bs in the present study remained almost fully activity after incubation at pH 2.0–13.0 for 1 h. Previous studies have also indicated that nattokinases are stable at temperatures lower than 40 °C but lose all initial activity after 10 min at 60 °C or 1 h at 50 °C. A thermostable nattokinase purified from *B. subtilis* B12 was relatively stable below 50 °C, but nearly 70% of its enzyme activity was lost at 60 °C for 40 min. The recombinant NK-Bs in the present study retained more than 50 and 40% of its enzymatic activity after incubation for 30 min at 60 °C and 65, respectively In addition, the present study also demonstrated that the recombinant NK-Bs had obvious thrombolytic activity, demonstrating that NK-Bs prepared with *P. pastoris* could be applied to health promotion. Altogether, the present study provides an ideal platform for the large-scale preparation of nattokinase at low cost.

## Conclusions

In this study, we achieved high levels expression of nattokinase from *B. subtilis* natto (NK-Bs) using a *Pichia pastoris* heterologous expression system and assess its fibrinolytic activity in vivo. Multicopy expression strains bearing 1–7 copies of the *apr*N gene were constructed. The expression level of the target protein reached a maximum at five copies of the target gene. However, multicopy expression strains were not stable in shake-flask or high-density fermentation, causing significant differences in the yield of the target protein among batches. Therefore, *P. pastoris* bearing a single copy of *apr*N was used in shake-flask and high-density fermentation. Target protein yield was 320 mg/L in shake-flask fermentation and approximately 9.5 g/L in high-density fermentation. The recombinant nattokinase showed high thermo- and pH-stability. The present study also demonstrated that recombinant NK-Bs had obvious thrombolytic activity.

## Materials and methods

All methods were carried out in accordance with relevant guidelines and regulations.

All experimental protocols were approved by Zhongnan Hospital of Wuhan University.

### Strains, plasmids, and media

*E. coli* XL10-Gold and *P. pastoris* GS115 were purchased from Invitrogen (USA). The vector pUC57 was purchased from GenScript (USA), and pHMB905BDM (d1 + 2 × 201 *AOX*1 promoter [29], MF4I signal sequence, Amp^r^) was constructed and stored in our lab. Luria-Bertani (LB) medium was prepared as described in the Manual of Molecular Cloning. Buffered glycerol-complex (BMGY) medium, buffered methanol-complex (BMMY) medium, and minimal dextrose (MD) medium were prepared as described in the instruction manual of the Invitrogen *Pichia* expression kit (USA) [29, 30]. BMMY medium supplemented with casein was prepared by dissolving 1% casein in phosphate buffer (pH 6.0), followed by the addition of BMMY ingredients and sterilization at 121 °C for 20 min. Lumbrokinase and fibrinogen were purchased from Sigma (USA).

### Design and synthesis of the truncated NK-Bs-encoding open reading frame

A truncated nattokinase-encoding gene from *B. subtilis* (NK-Bs) was used for heterologous expression. The DNA sequence of NK-Bs, excluding the first 52-bp signal sequence, was modified to match the codon usage bias of *P. pastoris* (S1). This edited open reading frame (ORF) was synthesized by Genecreate (China) and cloned into pUC57 for DNA sequencing.

### Construction of recombinant vectors for the expression of NK-Bs

The *apr*N gene of *B. subtilis* natto (Genbank accession number AEV91244.1) without the signal peptide coding sequence was optimized based on the codon preference of *P. pastoris* and synthesized. The DNA fragment was cloned into the expression vector pHBM905BDM to form an ORF encoding an NK-Bs protein fused with an N-terminal MF-4I leader sequence for secretory expression and a C-terminal 6× His-tag for affinity purification. It was amplified using the primers Natt-1 and Natt-2 (Table S1) and subsequently treated with T4 DNA polymerase in the presence of 1 mM deoxythymidine triphosphate (dTTP) for 20 min at 12 °C to form overhangs compatible with the sticky ends of pHBM905BDM digested with *Cpo*I and *Not*I [29, 30]. These two fragments were ligated with T4 ligase (TaKaRa, China), followed by transformation into *E. coli* XL10-Gold and screening on LB plates supplemented with 100 μg/mL ampicillin. Biobrick assembly was used for the construction of multicopy nattokinase expression vectors.

### Screening of recombinant yeast strains expressing NK-Bs

The recombinant plasmids were linearized using *Sal*I and transformed into *P. pastoris* GS115 by electroporation (7000 V/cm, 25 μF, 400×; Life Technologies Cell-Porator, USA). Transformants were selected on MD plates without histidine, followed by identification on BMMY plates supplemented with 1% casein [29, 30].

### Expression of recombinant NK-Bs using shake-flask fermentation

Recombinants bearing different copies of the target gene were inoculated into 50 mL BMGY medium and incubated at 28 °C for 2 days. The cells in each culture were collected by centrifugation at 4000×g for 5 min and individually inoculated into 30 mL BMMY medium. Then, 1% (v/v) methanol was added every 24 h to induce the expression of the foreign protein. Cell cultures were centrifuged at 10,000×g for 5 min at 4 °C after induction for 5 days, and the supernatants were collected.

### Sodium dodecyl sulfate-polyacrylamide gel electrophoresis and protein concentration measurements

Samples were separated using sodium dodecyl sulfate-polyacrylamide gel electrophoresis (SDS-PAGE) on a 12% (w/v) polyacrylamide gel, followed by staining with Coomassie Brilliant Blue G-250. Protein concentrations were determined using the Bradford method with a BCA protein kit (Pierce, USA). A standard curve was created using bovine serum albumin with concentrations ranging from 0.1 to 0.6 mg/mL [29, 30].

### Purification of the target protein

After induction with methanol for 120 h, the supernatant of the cell culture was collected and purified using Ni-NTA affinity chromatography. Approximately 20 mL of the supernatant was applied to 1 mL Ni-NTA beads. The column was washed twice with two column volumes of wash buffer (50 mM Tris-HCl; 200 mM NaCl; 50 mM NaH_2_PO_4_; 40 mM imidazole, pH 8.0). One column volume of elution buffer (50 mM Tris-HCl; 200 mM NaCl; 50 mM NaH_2_PO_4_; 200 mM imidazole, pH 8.0) was used to recover the target protein. The sample was then collected and dialyzed using a Millipore 10-kDa cut-off membrane at 4 °C to remove ions and salts, followed by resuspension in storage buffer (50 mM Tris-HCl, pH 7.5) [29, 30].

### Analysis of the serine proteinase activity of NK-Bs

Enzyme activity was measured using casein as the substrate. Briefly, 1 mL of diluted enzyme sample was mixed with an equal volume of 2% (w/v) casein in 20 mM phosphate buffer (pH 9.0), followed by incubation at 65 °C for 10 min. The reaction was terminated using 2 mL of 10% (w/v) trichloroacetic acid. The mixture was centrifuged at 14,000×g for 10 min and the optical density at 280 nm (OD_280_) of the supernatant was measured to determine the amount of tyrosine released during the reaction. One unit of enzymatic activity was defined as the amount of enzyme needed to catalyze the release of 1 μg tyrosine per min at 65 °C and pH 9.0. All experiments were performed in triplicate [29].

### Analysis of the characteristics of NK-Bs

To measure the optimal pH value for the recombinant enzyme, the pH of the enzyme solution was adjusted using the following buffers: sodium lactate (50 mM, pH 2.0–3.5); sodium acetate (50 mM, pH 3.5–6.0); phosphate buffer (50 mM, pH 6.0–7.0); Tris-HCl (50 mM, pH 7.0–9.0), or Na_2_B_4_O_7_/NaOH (50 mM, pH 9.0–12.0). The enzyme activity was then measured at 55 °C.

To determine the optimal temperature for the recombinant enzyme, the enzyme was diluted with Na_2_B_4_O_7_/NaOH buffer (50 mM, pH 10.5) to the appropriate concentration, and the reaction was carried out at a range of temperatures (50–80 °C, at 5 °C increments).

To investigate the thermostability of NK-Bs, the purified NK-Bs was divided into six samples and incubated at 50 °C, 55 °C, 60 °C, 65 °C, 70 °C, or 75 °C. Exactly 1 mL of each sample was collected every 10 min, and the remaining enzyme activity of all samples was measured at pH 9.0 and 65 °C.

To investigate the pH stability of NK-Bs, purified NK-Bs was incubated at pH 2–13 for 60 min, and the activity of the remaining enzyme was measured at pH 9.0 and 65 °C. Each experiment was performed in triplicate.

### Expression of recombinant NK-Bs using high-density fermentation

Fed-batch fermentation was performed according to the Invitrogen *Pichia* Fermentation Process Guidelines. The recombinant *P. pastoris* strain was inoculated into 200 mL YPD and cultivated at 28 °C for 24 h. The cell culture was then transferred to 2 L BSM medium in a 5-L fermenter (BaoXing, China). During the early stages of fermentation, the culture was maintained at 28 °C, pH 5.8, and 30% dissolved oxygen (DO). After approximately 18–24 h, glycerol was exhausted and DO was rapidly increased to 100%. To continue cell growth, 50% (v/v) glycerol supplemented with PTM trace salts (12 mL/L) was added at 12 mL/h/L, and DO was maintained above 20%. When the OD_600_ reached approximately 300, methanol containing PTM trace salts (12 mL/L) was fed at a speed of 3 mL/h/L to induce the expression of the target gene. The fermentation conditions were adjusted to 25 °C and pH 5.0, and DO was maintained at 20–30%. After 2 h, the feeding speed of methanol was increased by a ratio of 20% per hour until it reached 7 mL/h/L. These conditions were maintained until the end of fermentation [29, 30].

### Analysis of the fibrinolytic activity of NK-Bs in vitro

Fibrinolytic activity was measured using the standard fibrin plate method with modifications [31]. In a petri dish, 7.3 mL agarose (0.5% w/v) and 200 μL bovine thrombin (1 mg/mL) were gently mixed, followed by the addition of 2.5 mL bovine fibrinogen (1% w/v) to induce a solid fibrin formation. The fibrin plate was incubated at 37 °C for 18 h after 10 μL of the enzyme sample was injected into the bottom of the fibrin agar. The diameters of the clearing zones were used as indicators of the fibrinolytic activity of the enzyme.

### Analysis of the fibrinolytic effect of NK-Bs in vivo

To test the thrombolytic activity of recombinant NK-Bs in vivo, a rat model of microvascular thrombosis was established [32]. Female Sprague-Dawley rats weighing approximately 180–220 g were used. Thrombosis was induced by hypodermic injection of κ-carrageenan after the rats were fasted for 8 h. Thrombosis was evaluated by the length of tail infarction. The length of the infarcted region at the tip of the tail was measured 8, 12, and 24 h after injection. After the thrombosis model was established, the rats were split into six groups. Different dosages of the recombinant NK-Bs (50,000 IU/kg for high dose, 10,000 IU/kg for medium dose, and 2000 IU/kg for low dose) were fed to the rats every 12 h for 48 h. Vermis kinase, a known thrombolytic agent, was used as a positive control (10,000 IU/kg), and physiologic saline was used as the negative control. Each group included five duplicates.

Twelve hours after intragastric administration, the rats were anaesthetized by intraperitoneal injection with 50% ethyl carbamate at a dose of 5 mL/kg. Approximately 1.8 mL of blood from the postcaval vein was drawn into anticoagulant tubes containing 0.2 mL sodium citrate (0.109 mol/L). The blood in the tubes was centrifuged at 4000×g for 15 min, and 250 μL of the top layer of plasma was collected. Fibrin/fibrinogen degradation products (FDPs) and D-dimer levels were measured using the double antibody sandwich method (ABC-ELISA) according to the manufacturer’s protocol (Elabscience Biotechnology, China). Probabilities of less than 5% (*P* < 0.05) were considered statistically significant. All values are expressed as mean ± standard error of mean (S.E.M.) to show variation in groups.

### Histological examination

Histological examination was carried out as previously described [8, 9, 33]. Twelve hours after the nattokinase treatment was completed, rats were euthanized by spinal dislocation, and ear tissue was soaked in a formaldehyde solution before preparation of pathological sections. The tissue samples were embedded with paraffin and stained with hematoxylin and eosin for observation under a Upright optical microscope (Nikon Eclipse E100, Nikon DS-U3, 400).

## Supplementary Information

**Additional file 1: Fig. S1**. Analyzing the relation between expression levels of NK-Bs and the copy number of *apr*N gene with plate assay. **Table S1**. Primers for the amplification of ORF encoding NK-Bs. **Table S2**. The ratio of the opaque halo diameter to the colony diameter.

## Data Availability

The datasets supporting the conclusions of this article are included with in the article and its additional files. All strain materials were obtained from Hubei University, Wuhan, China.

## Abbreviations

NK-Bs: nattokinase from *B. subtilis* natto
MD: Minimal dextrose medium
BMMY: Buffered methanol-complex medium
BMGY: Buffered glycerol-complex medium
SDS-PAGE: sodium dodecyl sulfate-polyacrylamide gel electrophoresis
EDTA: ethylenediaminetetraacetic acid
PMSF: phenylmethylsulfonyl fluoride
FDP: Fibrin/fibrinogen degradation products
