# Supplementary material for: Heterologous expression of nattokinase from B. subtilis natto using Pichia pastoris GS115 and assessment of its thrombolytic activity
Source: BMC Biotechnol. 2021 Aug 9;21:49. doi: 10.1186/s12896-021-00708-4 (PMC8353737; doi:10.1186/s12896-021-00708-4)
Supplement: Supplementary file 1 — Additional file 1: Fig. S1. Analyzing the relation between expression levels of NK-Bs and the copy number of aprN gene with plate assay. Table S1. Primers for the amplification of ORF encoding NK-Bs. Table S2. The ratio of the opaque halo diameter to the colony diameter. [file 12896_2021_708_MOESM1_ESM.pdf]

Heterologous expression of nattokinase from *Bacillus subtilis* natto using *Pichia pastoris* GS115 and assessment of its thrombolytic activity

Yan Guangbo [1], Rao Ben[2], Shu Min [1], Shen Wei [1], Huang Zunxi [3], Ma Lixin [1], Zhai Chao [1*], Wang Yaping[1*]

1. State Key Laboratory of Biocatalysis and Enzyme, Engineering Hubei Collaborative Innovation Center for Green Transformation of Bio-Resources, Hubei Key Laboratory of Industrial Biotechnology, Biology Faculty of Hubei University, Hubei University , Wuhan, Hubei Province 430062, People's Republic of China

2. National Biopesticide Engineering Technology Research Center, Hubei Biopesticide Engineering Research Center, Hubei Academy of Agricultural Sciences, Biopesticide Branch of Hubei Innovation Centre of Agricultural Science and Technology, Wuhan, People's Republic of China

3. School of Life Sciences, Yunnan Normal University, Kunming, Yunnan, People's Republic of China

* Corresponding author: Wang Yaping; Zhai Chao. These two authors contributed equally to this work.

State Key Laboratory of Biocatalysis and Enzyme, Engineering Hubei Collaborative Innovation Center for Green Transformation of Bio-Resources, Hubei Key Laboratory of Industrial Biotechnology, Biology Faculty of Hubei University, Hubei University , Wuhan, Hubei Province 430062, People's Republic of China

Tel./fax: 027 88661237
E-mail address: 545080994 @qq.com

The optimized ORF encoding NK-Bs

atggctggtaagtcttctactgaaaagaagtacattgttggttttaagcagactatgtctgctatgagttctgctaagaagaagg
atgttatttccgaaaagggtggtaaggttcaaaagcaatttaagtacgttaatgctgccgctgctactttggatgaaaaggctg
ttaaggaattgaaaaaggacccctctgttgcttacgttgaagaagatcatattgctcatgaatacgctcaatctgttccatacgg
tatttctcaaatcaaggctccagctttgcattctcaaggttacaccggttcaaacgttaaggttgctgtgatagattctggtattg
atagttctcacccagatttgaacgttagaggtggtgcttcttttgtcccatctgaaactaacccataccaagatggctcctctcat
ggtactcatgttactggtactattgctgctttgaacaactctattggtgttttgggtgttgctccctctgcttcattgtacgctgttaa
agttttggattctactggttctggtcaatactcttggattattaacggtattgaatgggctatttctaacaacatggatgttattaac
atgtctttgggtggtccaactggctctactgctttgaagactgttgtcgataaggctgtttcttctggtattgttgttgctgcctctg
ctggtaacgaaggttcatctggttctacttctacggttggttacccagctaagtacccatctactattgctgttggtgctgttaact
cctctaaccaaagagcttctttttcttcagttggttctgaattggacgttatggcaccaggtgtttctattcaatctactttgccagg
tggtacttacggtgcttacaacggcacttctatggctactccacatgttgctggtgctgctgctttgattttgtctaagcatccaa
cttggactaacgctcaagttagagatagattggaaagtactgctacttacttgggtaactctttttactacggtaagggtttgatt
aacgttcaagcagccgcacaa

Supplementary Table1
Primers for the amplification of ORF encoding NK-Bs

Primer names	sequences
Natt-1	GTCAATGGCTGGTAAGTCTTCTACTGAAAAGAAGTACATTGTTGGTTTTAAG
Natt-2	GGCCATTATTGTGCGGCTGCTTGAACGTTAATCAAACCC

Note: the underlined Natt-1 and Natt-2 sequences matched the sticky ends generated by *Cpo*I and *Not*I, respectively.

Supplementary Fig. 1

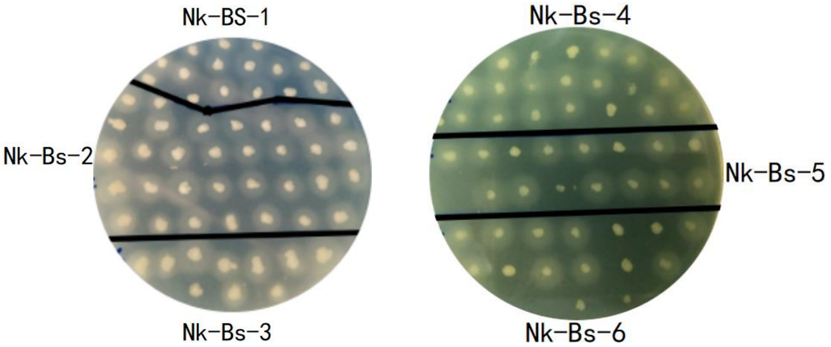

Fig. S1 analyzing the relation between expression levels of NK-Bs and the copy number of *apr*N gene with plate assay.

Supplementary Table 2
The ratio of the opaque halo diameter to the colony diameter

Strains	d_{oh}/d_c

NK-Bs-1	2.31±0.31
NK-Bs-2	2.92±0.28
NK-Bs-3	3.50±0.50
NK-Bs-4	4.36±0.44
NK-Bs-5	5.05±0.35
NK-Bs-6	4.85±0.48

Note: the average diameters of the colonies and halo were measured using plates in Fig. S1.
